# Growth and Development in Chinese Pre-Schoolers with Picky Eating Behaviour: A Cross-Sectional Study

**DOI:** 10.1371/journal.pone.0123664

**Published:** 2015-04-13

**Authors:** Yong Xue, Ai Zhao, Li Cai, Baoru Yang, Ignatius M. Y. Szeto, Defu Ma, Yumei Zhang, Peiyu Wang

**Affiliations:** 1 Department of Nutrition and Food Hygiene, School of Public Health, Peking University Health Science Center, Beijing, China; 2 Department of Social Medicine and Health Education, School of Public Health, Peking University Health Science Center, Beijing, China; TNO, NETHERLANDS

## Abstract

**Objective:**

To explore the associations between picky eating behaviour and pre-schoolers’ growth and development. Corresponding potential mechanisms, such as nutrient and food subgroup intake, as well as micronutrients in the blood, will be considered.

**Methods:**

Picky eating behaviour was present if it was reported by parents. From various areas of China, 937 healthy children of 3-7 years old were recruited using a multi-stage stratified cluster sampling method. Children and their mothers’ socio-demographic information and children’s anthropometry, intelligence, blood samples, one 24-hour dietary intake record and food frequency questionnaire were collected. Z-scores and intelligence tests were used to evaluate growth and development (cognitive development). Multilevel models were employed to verify the associations between picky eating behaviour and growth and development.

**Results:**

The prevalence of picky eating as reported by parents was 54% in pre-schoolers. Compared with the non-picky eaters, weight for age in picky eaters was 0.14 z-score (95% CI: -0.25, -0.02; *p* = 0.017) lower while no significant difference was found in intelligence (*p* > 0.05). Picky eating behaviour lasting over two years was associated with lower weight for age, as was nit-picking meat (the prevalence from parents’ perception was 23% in picky eaters) (*p* < 0.05). Picky eaters consumed fewer cereals, vegetables, and fish (*p* < 0.05), and had a lower dietary intake of protein, dietary fibre, iron, and zinc (*p* < 0.05). There were no differences in the concentrations of essential minerals in whole blood (*p* > 0.05).

**Conclusions:**

Picky eating behaviour is reported by parents in half of the Chinese pre-schoolers, which is negatively associated with growth (weight for age). Lower protein and dietary fibre as well as lower iron and zinc intakes were associated with picky eating as were lower intakes of vegetables, fish and cereals.

## Introduction

During the time of scarcity in China, social resources and family life were devoted to farming, securing, procuring and preparing food, which was limited in variety, had poor palatability, and low contents of energy and nutrients. With the rapid increase of comparative wealth and general affordability of food, parents are paying more attention to the diversity and composition of foods in children’s diets [1, 2]. Picky eating is often characterised as eating limited amounts of food, rejecting some specific subgroups of food, being unwilling to try new foods, and having strong preferences for certain cooking methods regarding the smell and taste of food [1, 3]. Picky eating results in limited and imbalanced composition of the diet and lower diversity of food intake [3–7]. Specifically in children [8–10] with a lower intake of vegetables and fruit, and a higher consumption of unhealthy processed foods, picky eating behaviour may be of considerable concern to parents. This behaviour forms a common complaint at physician visits and creates conflicts in feeding practice [7].

Picky eaters have a lower intake of food groups rich in micronutrients [6], such as fruits, vegetables, and meats, and a lower intake of vitamin C, vitamin E, fibre, and folate [1, 11, 12], which might result in a potential risk for nutritional deficits and inadequate weight. However, whether or not picky eating behaviour significantly stunts children’s growth and development is still a controversial topic. Some studies [2, 13, 14] focusing on toddlers with eating problems found that problematic eaters had long-standing problems with associated failure to thrive. Other studies [3, 11] reported that pre-schoolers with picky eating behaviour were more likely to be underweight and less likely to be overweight. However, there were also some studies [7, 15] showing no significant associations between picky eating behaviour and children’s growth, even when evaluated with z-scores.

Nutrition during early life may have a long-lasting influence on children’s cognitive development due to continuous development of frontal lobes throughout early childhood, especially for the first two years of life [16–18]. Pre-schoolers with picky eating starting in early life might suffer poor cognitive development. However, there is no clear conclusion on whether long-term unhealthy dietary habits, such as picky eating, have negative effects on children’s cognitive development.

Evidence [10, 19] is well documented suggesting a need for increasing the consumption of fruits and vegetables, supporting the recommendation of consuming more servings of fruits and vegetables as part of healthy dietary patterns for children. Previous studies [5, 6, 20] reported that picky eaters usually had limited dietary variety characterised by a lower intake of fruits, vegetables, and meats. Fruit and vegetable consumption might help children maintain a healthy weight by replacing excess energy derived from fatty foods [21]. However, the association between the lower intake of fruits and vegetables and growth (especially for the underweight) is unclear. A previous study [11] found that picky eating was associated with a lower intake of micronutrients such as calcium and magnesium; however, it is still unclear whether picky eating behaviour results in a lower content of micronutrients in blood. Moreover, there is limited knowledge of the eating behaviours of Chinese pre-schoolers.

Therefore, our research was conducted to estimate the proportion of pre-schoolers’ picky eating behaviour in China, to investigate possible associations between picky eating behaviour and children’s growth and development (including weight, height, BMI, intelligence, and z-scores about weight, height, and BMI), and to identify corresponding potential mechanisms, such as nutrient and food subgroup intake, as well as micronutrient levels in the blood.

## Subjects and Methods

### Study sample

The study protocol was reviewed and approved by the Ethical Committee of the Health Science Center at Peking University (NO.IRB00001052-11042). Written informed consent forms were signed by the legal guardians of all participants.

Data was collected between November 2011 and April 2012 from healthy pre-school children in China. By a using multi-stage stratified cluster sampling method, 937 children aged 3–7 years were enrolled in our study. In the first stage, according to geography and economic development level, seven cities (Beijing, Guangzhou, Chengdu, Shenyang, Suzhou, Lanzhou and Zhengzhou city), one village in the plains and one village in the mountainous area (both located in Xingtai, Hebei province), were selected *via* purposive sampling. In the second stage, in view of sample size and representativeness, one large kindergarten located in the semi-urban area of each city, and one large kindergarten in each village were selected. In the last stage, one junior-, one middle- and one senior-class in each kindergarten were selected randomly, and all children in the selected classes were surveyed. Criteria for eligibility were pre-schoolers aged 3–7 years, with no reported birth defects (including congenital heart disease, hydrocephalus or deformity at birth), no reported infantile paralysis and thalassemia, and no acute health problems (including common cold and diarrhoea) at present.

### Socio-demographics, anthropometry, intelligence, and blood measurement

The structured questionnaire was used to collect socio-demographic information through interviewing pre-schoolers’ parents, including child’s date of birth, gender, ethnicity, birth weight, dietary behaviour, and feeding patterns during the first four months after birth. Socio-demographic information about their parents was also collected, including date of birth, height, weight, educational level, and household income.

In every kindergarten, two well-trained investigators carried out the measurement of height and weight, respectively. Participants were asked to remove all heavy clothes and shoes (leaving only minimal clothing on in a comfortable preparation area), and weight was taken to the nearest 0.01 kg on a calibrated electronic weighing scale. Standing height was taken to the nearest to 0.1 cm using a height measuring tape suspended from the wall. The participants were required to stand erect with their shoulders level, hands by their sides, thighs together, and heels comfortably together, with the upper back, buttocks, and heels in contact with the wall during height measurement. All physical measurements were carried out three times, and the mean values were calculated. Intelligence was assessed in a quiet, isolated room using the Chinese version of the Wechsler Intelligence Scale for Children (C-WISC), which contains verbal and performance subtests. The Verbal (VIQ) and Performance (PIQ) scores from the Wechsler Intelligence Scale are composites of skills for performing tasks in the subtests, and all subtests are combined to produce a Full Scale IQ (FIQ). The C-WISC has shown good reliability and validity, and was used widely to measure pre-schoolers’ general intelligence in China [22–25]. Eight interviewers trained by a psychologist for children from Beijing Normal University administered the tests.

Fasting blood samples for the evaluation of microelements were collected in the morning. The blood samples were drawn *via* venepuncture, and were collected into 5-mL, metal free EDTA-treated tubes. During the process of blood collection, blood samples were kept on ice, occasionally shaken, then transferred to storage at 4°C. Calibration solution of the multi-minerals quality product in whole blood was obtained for quality assurances (BOHUI INNOVATION Ltd., Beijing). The concentration of calcium, copper, iron, magnesium, and zinc in blood were measured with a BOHUI 5100S atomic absorption spectrophotometer (BOHUI INNOVATION Ltd., Beijing) using hollow cathode lamps (422.7, 285.2, 248.3, 213.9, and 324.7 nm for calcium, magnesium, iron, zinc, and copper, respectively). All analyses on whole blood were performed in the same laboratory.

### Variables for picky eating

In the current study, picky eaters were characterised as children who consumed an inadequate variety and amount of food through rejection of foods that are familiar (and unfamiliar) to them because of specific tastes, textures, smells or appearances. The definition was provided to caregivers before the picky eating assessment, and caregiver’s perceptions were used to judge if their child was a “picky eater”. At each assessment in the structured questionnaire, the primary caregiver (usually the mother) was asked “Do you consider your child as having picky eating behaviour at present?” by choosing from three possible answers: 1) never picky, 2) somewhat picky, and 3) always picky. This single question was chosen based on observations from previous studies [5, 6, 26]. Children of those caregivers responding “somewhat” and “always” were categorised as picky eaters. Unless they answered “never”, they were asked to describe perceptions of the age of onset and duration of picky eating behaviour, and the specific foods nit-picked by picky eaters with response choices of milk, soy, cereal, vegetables, fruits, meat, and eggs.

### Dietary measurement

The nutrient intake of the pre-schoolers was determined from the dietary data collected using a 24-hour food intake record/recall. One trained, experienced researcher interviewed the mothers and another trained researcher checked the quality of food record/recall during the dietary measurement. Dietary data was collected at both the kindergarten and individual levels. Food consumption in the kindergarten was determined by conducting a detailed examination of changes in inventory before and after each meal with a kitchen scale. The individual nutritional intake was determined from the dietary data collected using a 24-hour food intake record, which was finished by researchers and kindergarteners. In consideration of some pre-schoolers having extra meals, the parents were required to record the extra food intake at home for 24 hours. To identify and quantify the food more accurately, some food models and a series of pictures of standard bowls and spoons were shown and explained to the parents before the dietary survey. The parents were asked to report all of the food their children consumed at home and while away from home. The nutrient contents of the foods were estimated using the China Food Composition book (2004) [27]. If their child was taking dietary supplements, the parents were asked to provide specific information regarding the supplements including brand names, manufacturers, and daily dosages in order to estimate the composition and content of nutrients. The FFQ (Food Frequency Questionnaire) was used to obtain data on consumption of the following food subgroups: cereals, vegetables, fruits, meat, fish, eggs, milk, nuts and beans, and oils and fats. Parents were asked to define the frequency and quantity of each subgroup of food consumed by their children over the last six months.

### Statistical analysis

The database was established using Epi Data version 3.0, and a double data entry was carried out. We used mean value ± standard error or percentage to describe the baseline characteristics of participants. Comparison of categorical data was carried out by using Pearson’s χ^2^ test, and a Student’s t-test was used for normally distributed data. According to the WHO Growth Standards, we calculated z-scores for height, weight, and BMI by using WHO AnthroPlus. To verify the functional relation between picky eating and growth and development of the pre-schoolers, to characterise the associations between the duration of picky eating and pre-schoolers’ growth and development, and to estimate the associations between the subgroups of food nit-picked and growth and development we used the statistical software MLwiN 2.31 to construct multilevel (two levels) mixed-effects linear regression models with investigation areas (level 1) nested within individuals (level 2). The multilevel model accounts for the clustering of the data; it can correct biases in parameter estimates and can produce correct CIs and significance tests. The regression coefficients were expressed by β, SEM and the 95% CI in comparison with the non-picky eating group. All of the correlation analyses for height, weight, and BMI were adjusted for the following variables: age, gender, and birth weight of the children, education of the mothers, income per capita of the families, and the feeding patterns during the first four months after birth. All of the correlation analyses of z-scores were also adjusted for variables listed above (not including age and gender). As for nutrient intake, intake of food groups and mineral content in whole blood, mean value ± standard error was used to describe continuous variables, and Student’s t-tests and covariance analyses adjusted for child’s gender and age were used to compare the differences between picky eaters and non-picky eaters. All t-tests and covariance analyses were performed using SPSS 15.0. Statistical significance was determined at *p* < 0.05 (two-tailed tests).

## Results

At the initial interview, 937 healthy pre-schoolers participated in our survey. Only those pre-schoolers with their anthropometry and intelligence measured, and who completed their assessment of picky eating behaviour, were included in this study, resulting in the final analysis set of 911 eligible pre-schoolers including 423 (46%) non-picky eaters and 488 (54%) picky eaters. Socio-demographic information of the children and their mothers is provided in Table 1. There were no significant differences in height, weight, and BMI of the mothers, or in the household income between the picky eating and non-picky eating groups. The mothers in the picky eating group were younger (*p* < 0.05) and had lower levels of education (*p* = 0.004). The group of picky eaters did not differ from the non-picky eating group in birth weight, ethnicity, gender, and feeding patterns during the first four months after birth. However, the pre-schoolers in the picky eating group were younger (*p* = 0.002).

**Table 1:** Socio-demographic characteristics of mother-pre-schooler dyads of the subjects.

	Non-picky eating (n = 423)		Picky eating (n = 488)		*p* value
Mother's characteristics					
	N	Mean ± SE ^b^	N	Mean ± SE ^b^	
Age, y ^a^	416	32.48 ± 0.21	476	31.87 ± 0.20	0.036
Height, cm	420	161.32 ± 0.23	483	161.19 ± 0.22	0.678
Weight, kg	412	55.65 ± 0.36	472	56.07 ± 0.35	0.402
BMI, kg/m^2^	412	21.38 ± 0.13	472	21.57 ± 0.13	0.288
		N (%)		N (%)	
Education ^a^					0.004
Middle school or below		144 (34)		167 (34)	
High school		89 (21)		142 (29)	
College or above		184 (44)		165 (34)	
Unclear		6 (1)		14 (3)	
Family’s per capita income, Yuan/mo					0.795
< 2000		148 (35)		183 (38)	
2000–4000		118 (28)		127 (26)	
> 4000		79 (19)		95 (19)	
Unknown		78 (18)		83 (17)	
		Mean ± SE ^b^		Mean ± SE ^b^	
Child's characteristics					
Age, y ^a^	423	4.94 ± 0.05	488	4.76 ± 0.04	0.002
Birth weight, kg	407	3.37 ± 0.03	477	3.36 ± 0.02	0.794
		N (%)		N (%)	
Ethnicity					0.302
Han		411 (97)		468 (96)	
Others		12 (3)		20 (4)	
Gender					0.616
Male		214 (51)		255 (52)	
Female		209 (49)		233 (48)	
Feeding pattern during the first four months after birth					0.205
Exclusive breastfeeding		204 (48)		256 (52)	
Mixed feeding		157 (37)		159 (33)	
Artificial feeding		58 (18)		72 (15)	
Unclear		4 (1)		1 (0)	

^a^ indicates significant differences between non-picky eating and picky eating groups, *p* < 0.05.

^b^ SE = standard error.

The results of correlations between picky eating behaviour and the growth and development of children are provided in Table 2. Crude and adjusted linear regression analyses indicated that children in the picky eating group had significantly lower weights and z-scores of weight for age compared with the non-picky eating group. Picky eating was associated with a 0.42 kg difference (lower) in weight (95% CI: -0.72, -0.12; *p* = 0.006) and a 0.14 unit lower in the z-score of weight for age (95% CI: -0.25, -0.02; *p* = 0.017), respectively, compared with non-picky eating. Though significant differences in height, z-score of height for age, and intelligence were found in picky eating and non-picky eating groups (*p* < 0.05), there were no significant differences between the two groups after adjusting for other influencing factors (*p* > 0.05). In the current study, a crude analysis indicated that children in the non-picky eating group did not have significantly lower BMIs and z-scores of BMIs for age, compared with the picky eating group (*p* < 0.05). Though significant differences in BMIs were found between the two groups according to the adjusted linear regression (*p* < 0.05), there was no significant difference in z-scores of BMIs for age from the linear model (*p* > 0.05).

**Table 2:** Parameters of growth and development of non-picky eating and picky eating groups.

	Non-picky eating ^a^ (n = 423)	Picky eating ^a^ (n = 488)	*p* value	Adjusted ^†^ β ^‡^	SEM	95% confidence interval	Adjusted *p* value
Height, cm	110.45 ± 0.39	108.66 ± 0.33	< 0.001	-0.41	0.28	-0.96, 0.14	0.141
Height for age	0.31 ± 0.05	0.18 ± 0.04	0.046	-0.10	0.06	-0.22, 0.02	0.087
Weight, kg	18.96 ± 0.16	18.11 ± 0.13	< 0.001	-0.42	0.15	-0.72, -0.12	0.006
Weight for age	0.23 ± 0.05	0.08 ± 0.04	0.016	-0.14	0.06	-0.25, -0.02	0.017
BMI, kg/m^2^	15.46 ± 0.07	15.28 ± 0.06	0.061	-0.21	0.09	-0.38, -0.04	0.016
BMI for age	0.04 ± 0.05	-0.06 ± 0.04	0.102	-0.12	0.06	-0.23, 0.00	0.056
Intelligence, IQ	100.65 ± 0.72	98.57 ± 0.62	0.026	-0.79	0.80	-2.36, 0.77	0.321

^a^ Values were given as Mean **±** SE.

^†^ All of the models were constructed by using multilevel (two levels) mixed-effects liner regression with the iterative generalised least-squares estimation method. Results of the measurements from the regression models with adjustment for child’s age, gender (female, male), birth weight, and feeding pattern during the first four months after birth (exclusive breastfeeding, mixed feeding, artificial feeding, and unclear), mother’s education (middle school or below, high school, college or above, and unclear), and family’s per capita monthly income (< 2000, 2000–4000, > 4000, and unclear, Yuan). Results of z-scores from the regression models with adjustment for child’s birth weight and feeding pattern during the first four months after birth, mother’s education, and household income.

^‡^ β represents the difference in mean parameters of growth and development between non-picky eating and picky eating groups after adjusting for the covariates listed above.

The picky eaters had different durations of picky eating behaviour: picky eating lasted 0–2 years in 203 (42%) subjects, 2–3 years in 124 (25%) subjects, and over 3 years in 104 (21%) subjects. To further verify the associations between picky eating behaviour and pre-schoolers’ growth and development, comparisons between non-picky eating and picky eating groups with different durations were carried out (Table 3). The z-scores of height for age, weight for age, and BMI for age decreased with an increase in the duration of picky eating behaviour. Compared with the non-picky eating group, there were no significant differences in z-scores of height for age, weight for age and BMI for age during the first and second year of picky eating (*p* > 0.05). Compared with the corresponding values in the non-picky eating group, lower z-scores of weight for age were found in pre-schoolers with picky eating lasting over two years, and lower z-scores of BMI for age were found in those with picky eating for over three years by crude and adjusted linear regression analyses (*p* < 0.05). Picky eating lasting 2–3 years and over three years were associated with 0.22 (95% CI: -0.39, -0.05; *p* = 0.011) and 0.25 (95% CI: -0.43, -0.06; *p* = 0.008) z-scores lower in weight for age, respectively. Being a picky eater for over three years was associated with a 0.10 (95% CI: -0.45, -0.06; *p* = 0.012) z-score lower in BMI for age. Compared with the non-picky eating group, there were no significant differences in intelligence of pre-schoolers with picky eating lasting any years (*p* > 0.05).

**Table 3:** Z-scores for height, weight, and BMI of pre-schoolers with picky eating habits of different durations, and adjusted associations between z-scores and duration of picky eating behaviours.

	N	Mean ± SE ^a^	Unadjusted *p*	Adjust ^†^ β ^‡^	SEM	95% CI ^b^	Adjusted *p*
Height for age							
Non-picky eating	423	0.31 ± 0.05	Reference				
0–2, y	203	0.22 ± 0.06	0.287	-0.09	0.08	-0.25, 0.06	0.223
2–3, y	124	0.10 ± 0.10	0.039	-0.17	0.09	-0.35, 0.01	0.069
>3, y	104	0.03 ± 0.08	0.008	-0.15	0.10	-0.35, 0.04	0.120
Unclear	57	0.47 ± 0.11	0.252	0.10	0.13	-0.15, 0.35	0.423
Weight for age							
Non-picky eating	423	0.23 ± 0.05	Reference				
0–2, y	203	0.15 ± 0.06	0.294	-0.05	0.07	-0.19, 0.09	0.504
2–3, y	124	0.01 ± 0.08	0.019	-0.22	0.09	-0.39, -0.05	0.011
>3, y	104	-0.08 ± 0.09	0.002	-0.25	0.09	-0.43, -0.06	0.008
Unclear	57	-0.28 ± 0.11	0.685	-0.06	0.12	-0.29, 0.18	0.647
BMI for age							
Non-picky eating	423	0.04 ± 0.05	Reference				
0–2, y	203	0.00 ± 0.07	0.601	0.01	0.08	-0.14, 0.16	0.888
2–3, y	124	-0.09 ± 0.08	0.187	-0.17	0.09	-0.36, 0.01	0.062
>3, y	104	-0.17 ± 0.10	0.043	-0.25	0.10	-0.45, -0.06	0.012
Unclear	57	-0.03 ± 0.13	0.582	-0.20	0.13	-0.45, 0.05	0.124
Intelligence, IQ							
Non-picky eating	423	100.65 ± 0.72	Reference				
0–2, y	203	98.03 ± 0.94	0.032	-0.50	1.05	-2.56, 1.57	0.639
2–3, y	124	99.04 ± 1.23	0.267	-1.15	1.22	-3.53, 1.23	0.345
>3, y	104	99.72 ± 1.33	0.548	-0.42	1.32	-3.01, 2.17	0.751
Unclear	57	97.37 ± 1.98	0.101	-1.53	1.70	-4.86, 1.79	0.366

^†^ All of the models were constructed by using multilevel (two levels) mixed-effects liner regression with the iterative generalised least-squares estimation method. Results of the z-scores from the regression models with adjustment for child’s birth weight and feeding pattern during the first four months after birth, mother’s education, and family’s per capita monthly income. Results of intelligence from the regression models with adjustment for child’s age, gender, birth weight, and feeding pattern during the first four months after birth, mother’s education, and family’s per capita monthly income.

^‡^ β represents the difference in mean z-scores between the non-picky eating group and the picky eating group after adjusting for the covariates listed above.

^a^ SE = standard error,

^b^ CI, confidence interval.

The food nit-picked by picky eaters were mostly vegetables (56% of the picky eaters), meat (23%), soy (21%), eggs (21%), cereal (19%), fruits (12%), and milk (8%). Children nit-picking of meat had a 0.19 (95% CI: -0.37, -0.02; *p* = 0.034) z-score lower in weight for age and a 0.23 (95% CI: -0.41, -0.04; *p* = 0.017) z-score lower in BMI for age. No significant associations were found between z-scores and nit-picking of vegetables, soy, eggs, cereal, fruits or milk (p >0.05) (Table 4).

**Table 4:** Influence of nit-picking of subgroups of food on growth and development of pre-schoolers.

		Height for age			Weight for age			BMI for age		
	N	Adjusted ^†^ β ^‡^ (95% CI ^a^)	SEM	Adjusted *p*	Adjusted ^†^ β ^‡^ (95% CI ^a^)	SEM	Adjusted *p*	Adjusted ^†^ β ^‡^ (95% CI ^a^)	SEM	Adjusted *p*
Milk	37	-0.09 (-0.39, 0.22)	0.15	0.570	-0.19 (-0.47, 0.09)	0.14	0.185	-0.23 (-0.53, 0.07)	0.15	0.138
Soy	104	-0.06 (-0.26, 0.13)	0.10	0.527	-0.12 (-0.30, 0.06)	0.09	0.120	-0.13 (-0.32, 0.07)	0.10	0.201
Cereal	92	-0.10 (-0.30, 0.11)	0.10	0.348	-0.02 (-0.21, 0.17)	0.10	0.840	0.06 (-0.14, 0.26)	0.10	0.544
Vegetables	274	-0.01 (-0.12, 0.14)	0.07	0.882	0.01 (-0.12, 0.13)	0.06	0.882	0.01 (-0.14, 0.13)	0.07	0.909
Fruits	58	-0.07 (-0.32, 0.19)	0.13	0.612	-0.03 (-0.27, 0.20)	0.12	0.786	0.01 (-0.24, 0.26)	0.13	0.920
Meat	110	-0.06 (-0.25, 0.13)	0.10	0.540	-0.19 (-0.37, -0.02)	0.09	0.034	-0.23 (-0.41, -0.04)	0.10	0.017
Eggs	101	-0.14 (-0.34, 0.05)	0.10	0.152	-0.12 (-0.30, 0.07)	0.09	0.212	-0.05 (-0.25, 0.14)	0.10	0.603

^†^ All of the models were constructed by using multilevel (two levels) mixed-effects liner regression with the iterative generalised least-squares estimation method. Results of the z-scores from the regression models with adjustment for child’s birth weight and feeding pattern during the first four months after birth, mother’s education, family’s per capita monthly income and other kinds of food related with children’s growth listed above.

^‡^ β represents the difference in mean z-scores between the non-picky eating group and the picky eating group after adjusting for the covariates listed above.

^a^ CI, confidence interval.

The results of dietary mean nutrient intake from the 24-hour dietary recall are shown in Table 5. Compared with the non-picky eating group, the intake of protein and dietary fibre was lower in the picky eating group according to the crude and adjusted linear regression analyses (*p* < 0.05). There was no significant difference in intake of energy, fat and carbohydrates or vitamins between the two groups (*p* > 0.05). The crude and adjusted linear regression analyses showed a lower intake of iron and zinc (*p* < 0.05), but no differences in the intake of magnesium, calcium, and copper (*p* > 0.05).

**Table 5:** Dietary intake of energy, macronutrients, dietary fibre, minerals and vitamins of pre-schoolers of non-picky eating and picky eating groups.

	Non-picky eating		Picky eating		*p* value	
	Mean	SE ^e^	Mean	SE ^e^	Unadjusted	Adjusted ^†^
Energy (kcal)	1627.65	34.65	1554.05	34.27	0.133	0.232
Protein ^a^ (g)	55.77	1.37	51.81	1.10	0.023	0.038
Fat (g)	59.29	1.31	57.82	1.48	0.461	0.488
Carbohydrate (g)	225.11	5.77	213.77	5.17	0.143	0.300
Dietary fibre ^a^ (g)	7.61	0.28	6.83	0.21	0.027	0.049
Vitamin A (μgRE ^b^)	481.72	26.51	543.14	42.79	0.240	0.385
Thiamine (mg)	0.81	0.03	0.87	0.07	0.440	0.466
Riboflavin (mg)	0.93	0.05	0.91	0.05	0.774	0.714
Niacin (mgNE ^c^)	11.18	0.32	10.97	0.32	0.641	0.790
Vitamin C (mg)	63.95	2.42	66.70	3.21	0.505	0.715
Vitamin E (mgα-TE ^d^)	19.08	0.48	19.14	0.60	0.941	0.820
Calcium (mg)	443.05	22.87	446.17	24.42	0.926	0.975
Magnesium (mg)	230.54	6.72	210.49	5.35	0.020	0.059
Iron ^a^ (mg)	17.29	0.52	15.67	0.41	0.014	0.038
Zinc ^a^ (mg)	9.33	0.45	8.31	0.24	0.044	0.046
Copper ^a^ (mg)	1.48	0.04	1.62	0.09	0.128	0.157

^a^ indicates significant differences between non-picky eating and picky eating groups (*p* < 0.05).

^b^ Retinol equivalent;

^c^ Niacin equivalent;

^d^ α-Tocopherol equivalent;

^e^ SE = standard error.

^†^ Results of nutrition intake from covariance analysis with adjustment for child’s gender and age.

The mean intake of food groups (g/day) in the non-picky eating and picky eating groups are shown in Table 6. According to crude and adjusted linear regression analyses, picky eaters had a lower dietary intake of cereals (*p* < 0.05), vegetables (*p* < 0.001) and fish (*p* < 0.05). However, there were no significant differences in the intake of fruit, meat, eggs, milk, nuts and beans, or oils and fats (*p* > 0.05) between the two groups.

**Table 6:** Comparison of intake (g/day) of various groups of food between pre-schoolers in non-picky and picky eating groups.

	Non-picky eating		Picky eating		*p* value	
	Mean	SE ^b^	Mean	SE ^b^	Unadjusted	Adjusted ^†^
Cereals ^a^	273.69	8.35	244.77	6.88	0.007	0.035
Vegetables ^a^	204.26	8.74	154.46	6.33	< 0.001	< 0.001
Fruits	183.14	7.62	167.86	7.45	0.153	0.114
Meat	83.19	3.91	75.25	3.77	0.146	0.091
Fish ^a^	63.19	4.33	50.30	3.87	0.027	0.013
Eggs	71.80	2.37	68.15	3.12	0.364	0.402
Milk	228.90	10.42	210.23	9.26	0.179	0.177
Nuts and beans	79.96	5.23	73.08	4.07	0.294	0.311
Oils and fats	22.73	1.32	22.86	1.46	0.946	0.911

^a^ indicates significant differences between non-picky eating and picky eating groups, *p* < 0.05.

^b^ SE = standard error.

^†^ Results of food intake from covariance analysis with adjustment for child’s gender and age.

The micronutrients found in whole blood are summarised in Table 7. We found no difference between the two groups (*p* > 0.05).

**Table 7:** Comparison of the micronutrient content in whole blood of pre-schoolers in non-picky and picky eating groups.

	Non-picky eating		Picky eating		*p* value
	Mean	SE ^a^	Mean	SE ^a^	
Calcium (mmol/L)	1.754	0.007	1.747	0.006	0.470
Magnesium (mmol/L)	1.447	0.007	1.451	0.007	0.649
Iron (mmol/L)	7.019	0.039	7.689	0.037	0.831
Zinc (μmol/L)	76.123	0.818	77.563	0.810	0.213
Copper (μmol/L)	15.470	0.151	15.641	0.148	0.422

^a^ SE = standard error.

## Discussion

We found that parents perceived 54% of their Chinese pre-schoolers to be picky eaters; the children consumed low quantities of limited diversity. Nit-picking, mostly vegetable, was reported in 56% of picky eaters. Picky eaters had younger, lower educated mothers. Growth was associated with picky eating: weight for age was lower and BMI as a consequence also, especially if the duration of picky eating was longer than 2 years; however, cognitive development was not. Dietary intake of picky eaters was lower in protein and fibre, as well as iron and zinc; consumption of vegetables, cereals and fish was lower. Whole blood values of minerals were unaffected. Nit-picking meat was associated with lower weight for age and BMI for age, but not with height for age. Nit-pickers for meat consumed fewer meat, cereals, vegetables, and eggs (S3 Table), and had a lower dietary intake of magnesium (S2 Table), while whole blood values of minerals were unaffected (S4 Table).

Previous studies [6, 28–31] have reported children’s food preferences, which are developed from genetically determined predispositions and experiences, such as repeated exposure, feeding context, and social and physiological consequences, are the consistent determining factor of food acceptance. The first few years of life are considered a critical period for the development of food acceptance patterns [32, 33] due to early exposure to flavours through breastfeeding [34, 35], artificial feeding [36], and complementary feeding [37] in babyhood and the toddler period. Food preference shaped by repeated experiences during the pre-school period may further lead to stability. Previous studies [38–39] reported that eating problems, including picky eating, were demonstrated continuously from early childhood into mid-adolescence, which was related to both later eating disorders, lasting fussy eating, and a limited variety of food preferred in adolescence and adulthood.

Picky eating is considered to be a complex behaviour [7, 20, 26, 40], which is difficult to quantify accurately in childhood. Picky eaters are usually defined as children who consume an inadequate amount and variety of food through rejection of some foods [1, 41]. Our definition of picky eating behaviour was consistent with this. In our study, we found that picky eating behaviour was relatively common in Chinese pre-schoolers aged 3–7 years, and the proportion of picky eaters was up to 54%. Previous studies [3–5, 7, 20, 42, 43] reported the frequency of picky eating behaviour with great variation, ranging from 8% to 50% of children, due to the different ages of children included, the varying areas/country of the study population, and the different definitions and assessment of picky eating [1]. In the present study, children who were picky eaters had younger mothers with lower levels of education than the non-picky eaters. Previous studies [44, 45] pointed out that parental control of feeding and eating patterns might contribute to children’s eating behaviour. Older and more educated mothers might pay more attention to their children’s eating behaviour and provide their children with a better selection of fruits and vegetables [46] resulting in much better eating habits.

Previous studies [3, 7, 15, 47] showed inconsistent findings regarding the associations between picky eating behaviour and growth and development in children older than three years. Dubois et al [3] found that picky eaters were twice as likely as non-picky eaters to be underweight at 4.5 years old, but Mascola et al [7] did not observe similar results in their prospective study. In another study, Wright et al [15] found weak associations between picky eating and poor growth, and simply eating a limited variety was found to be unrelated to growth. Carruth et al [47] followed 71 children with picky eating behaviour for 34–84 months and found that mean weights and heights were within normal values for age and genders. In the aforementioned studies, just a few results [3] were adjusted for confounding factors. Actually, children’s characteristics, such as age, gender, birth weight [48], and feeding practices [49] during early life, and parents’ characteristics, such as mother’s education and household income, played an important role in children’s growth and development. Other explanations for controversial results about picky eating may include inconsistent definitions and methods for the assessment of picky eating, lack of existing applicable measures, and the inappropriate comparison between the picky eating and non-picky eating groups.

In the present study, crude and adjusted linear regression analyses indicated significantly lower weights and z-scores of weight in the picky eating group than in the non-picky eating group. Previous studies [3–7] reported that picky eaters had lower food diversity and ingested limited amounts of food, such as fruits, vegetables, and meat [6], and a lower intake of calories [50], fat, calcium, magnesium, vitamin C, vitamin E [11], and folate [1, 12], which contribute to children’s growth. At the same time, a lower intake of protein and iron by picky eaters was found in the present study, which predisposed the child to infection, being underweight, and a stunting of the growth [51]. In addition, lower intelligence in the picky eating group was not detected in adjusted linear regression analyses, though lower zinc intake was found in the picky eating group. Studies [52] in animal models and humans demonstrated the effects of zinc deficiency on decreased cognitive development activities; thus, it is necessary to identify whether long-term lower zinc intake among picky eaters may result in zinc deficiency and impairment in cognitive development in the future.

In this study, over 45% of picky eaters had this behaviour for over two years, and only 14% of the children were recognised as picky eaters during the past year, which suggests that picky eating starts mainly during early childhood and is often of longer duration. A previous study [7] reported picky eaters of longer durations were less likely to accept new foods and showed stronger liking or disliking of food than those of shorter durations. To further verify the association between picky eating and growth and development in pre-schoolers, we compared the non-picky eating group with the picking eating group separated into duration of pickiness. In agreement with our hypothesis, z-scores for weight and BMI were significantly lower in children with picky eating habits lasting longer than three years.

Although a lower intake of some subgroups of food and nutrients was found in picky eaters (and picky eating behaviour might have negative effects on children’s growth), the associations between the intake of specific subgroups of food and the growth of children are still unclear. A previous study reported that replacing fresh produce, such as fruits and vegetables, with industrially processed foods might increase the risk of being overweight and having chronic diseases in the future [1]. On the other hand, nit-picking subgroups of food might result in children being underweight. In the present study, nit-picking meat was found to have lower z-scores for weight and BMIs. Meat, an important source of protein, fat, heme iron and fat-soluble vitamins, contributes to children’s nutritional status and growth. Appropriate intake of meat is very important for the nutritional needs of children. The general recommendation should be provided for picky eaters (just like the intake of vegetables and fruits: at least five portions each day for children [53]).

Galloway [11] found that picky eaters consumed fewer foods containing vitamin E, vitamin C, folate and fibre, probably due to their lower consumption of fruits and vegetables compared to non-picky eaters. In the present study, a lower intake of protein was found in the picky eating group, probably due to lower consumption of fish. Lower dietary intake of fibre, iron, and zinc were also found, most likely due to a lower intake of cereals, vegetables and fish. Considering the long-term lower intake of proteins and micronutrients may result in nutritional deficiencies, which may have long-term developmental consequences [54], considerable resources have been directed to the nutritional assessment [55] of the pre-school years when nutrient requirements are exacerbated by rapid growth. However, in our study, we did not find significant differences between picky eaters and non-picky eaters regarding the content of essential minerals in whole blood.

This study has several limitations. Firstly, it was not accurate enough to quantify picky eating behaviour in pre-schoolers by simply asking their parents whether or not they considered their child to be a picky eater. Differences in the judgment of picky eating exist in parents’ perceptions, which might affect data interpretation [1]. Although a strict definition was provided in our study, some children still might be misclassified because of subjective reports and measurement. Therefore, it is essential that a universal and widely applicable psychometric tool be offered to measure picky eating behaviour with valid and reliable measures for future research. Secondly, although significant associations between picky eating behaviour and children’s growth have been found, our cross-sectional study design did not provide direct evidence of the causality. A future prospective longitudinal study with a large sample size and consideration of potential confounders needs be carried out. Thirdly, the 24-hour dietary recall is widely used to assess children’s nutrition intake [5, 10, 11], but its variability results in difficulty in accurately estimating individual’s long-term diets [56]. In the present study, to reduce the influence of this limitation, a trained researcher interviewed parents and their child in a face-to-face interview with the aid of food models. Long-term dietary information from the FFQ was also collected, which contributed to the comprehensive understanding of children’s nutrient intake.

## Conclusions

Picky eating in Chinese pre-schoolers is very common, reported more often by younger mothers with lower education. It is associated with lower weight for age especially when lasting over two years. Lower intake of protein, fiber, iron, and zinc is found in these pre-schoolers, probably due to lower vegetables, cereal and fish intake. Nit-picking meat is associated with lower weight for age and BMI for age, and this relationship requires further investigation to disentangle the possible nutrimental, environmental, and hereditary causal pathways of these mechanisms. We therefore recommend standardization of picky eating and propose the definition we used. Parents should pay attention to nit-picking meat and picky eating in order to prevent their lasting effects on the growth of pre-schoolers.

## Supporting Information

S1 Dataset Database of the present study (RAR)

S1 Table Socio-demographic characteristics of mother-preschooler dyads of non-picky eaters and children nit-picking meat. ^a^ indicates significant differences between non-picky eating and picky eating groups, *p*<0.05. ^b^ SE = standard error.
(DOCX)

S2 Table Dietary intake of energy, macronutrients, dietary fibre, minerals and vitamins of pre-schoolers of non-picky eating and nit-picking meat groups. ^a^ indicates significant differences between non-picky eating and picky eating groups (*p* < 0.05). ^b^ Retinol equivalent; ^c^ Niacin equivalent; ^d^ α-Tocopherol equivalent; ^e^ SE = standard error. ^†^ Results of nutrition intake from covariance analysis with adjustment for child’s gender and age.
(DOCX)

S3 Table Comparison of intake (g/day) of various groups of food between pre-schoolers in non-picky eating and nit-picking meat groups. ^a^ indicates significant differences between non-picky eating and picky eating groups, *p* < 0.05. ^b^ SE = standard error. ^†^ Results of food intake from covariance analysis with adjustment for child’s gender and age.
(DOCX)

S4 Table Comparison of the micronutrient content in whole blood of pre-schoolers in non-picky and nit-picking meat groups. ^a^ SE = standard error.
(DOCX)
