# Supplementary material for: Growth and Development in Chinese Pre-Schoolers with Picky Eating Behaviour: A Cross-Sectional Study
Source: PLoS One. 2015 Apr 13;10(4):e0123664. doi: 10.1371/journal.pone.0123664 (PMC4395402; doi:10.1371/journal.pone.0123664)
Supplement: S1 Table — a indicates significant differences between non-picky eating and picky eating groups, p<0.05. b SE = standard error. (DOCX) [file pone.0123664.s002.docx]

**S 1 Table. Socio-demographic characteristics of mother-preschooler dyads of non-picky eaters and children nit-picking meat.**

|  | | | Non-picky eating  (n=423) | | Nit-picking meat  (n=110) | | *P*  value |
| --- | --- | --- | --- | --- | --- | --- | --- |
| Mother's characteristics | | |  | |  | |  |
|  |  | | N | Mean ± SE ^b^ | N | Mean ± SE ^b^ |  |
|  | Age, y | | 416 | 32.48 ± 0.21 | 108 | 31.55 ± 0.46 | 0.050 |
|  | Height, cm | | 420 | 161.32 ± 0.23 | 109 | 161.13 ± 0.47 | 0.706 |
|  | Weight, kg | | 412 | 55.65 ± 0.36 | 107 | 54.36 ± 0.75 | 0.110 |
|  | BMI, kg/m^2^ | | 412 | 21.38 ± 0.13 | 107 | 20.89 ± 0.24 | 0.079 |
|  |  | |  | N (%) |  | N (%) |  |
|  | Education | |  |  |  |  | 0.112 |
|  |  | Middle school or below |  | 144 (34) |  | 37 (34) |  |
|  |  | High school |  | 89 (21) |  | 33 (30) |  |
|  |  | College or above |  | 184 (44) |  | 37 (34) |  |
|  |  | Unclear |  | 6 (1) |  | 3 (3) |  |
|  | Family’s per capita income,  Yuan/mo (RMB) | |  |  |  |  | 0.675 |
|  |  | < 2000 |  | 148 (35) |  | 34 (31) |  |
|  |  | 2000 ~ 4000 |  | 118 (28) |  | 30 (27) |  |
|  |  | > 4000 |  | 79 (19) |  | 26 (24) |  |
|  |  | Unknown |  | 78 (18) |  | 20 (18) |  |
|  |  |  |  | Mean ± SE ^b^ |  | Mean ± SE ^b^ |  |
| Child's characteristics | | |  |  |  |  |  |
|  | Age, y ^a^ | | 423 | 4.94 ± 0.05 | 110 | 4.76 ± 0.08 | 0.002 |
|  | Birth weight, kg ^a^ | | 407 | 3.37 ± 0.03 | 106 | 3.24 ± 0.05 | 0.032 |
|  |  | |  | N (%) |  | N (%) |  |
|  | Ethnicity | |  |  |  |  | 0.302 |
|  |  | Han |  | 411 (97) |  | 468 (96) |  |
|  |  | Others |  | 12 (3) |  | 20 (4) |  |
|  | Gender | |  |  |  |  | 0.616 |
|  |  | Male |  | 214 (51) |  | 255 (52) |  |
|  |  | Female |  | 209 (49) |  | 233 (48) |  |
|  | Feeding pattern during the  first four months after birth | |  |  |  |  | 0.702 |
|  |  | Exclusive breastfeeding |  | 204 (48) |  | 51 (46) |  |
|  |  | Mixed feeding |  | 157 (37) |  | 38 (35) |  |
|  |  | Artificial feeding |  | 58 (14) |  | 20 (18) |  |
|  |  | Unclear |  | 4 (1) |  | 1 (1) |  |

^a^ indicates significant differences between non-picky eating and picky eating groups, *p*<0.05.

^b^ SE = standard error.
