# Supplementary material for: Growth and Development in Chinese Pre-Schoolers with Picky Eating Behaviour: A Cross-Sectional Study
Source: PLoS One. 2015 Apr 13;10(4):e0123664. doi: 10.1371/journal.pone.0123664 (PMC4395402; doi:10.1371/journal.pone.0123664)
Supplement: S2 Table — a indicates significant differences between non-picky eating and picky eating groups (p < 0.05). b Retinol equivalent; c Niacin equivalent; d α-Tocopherol equivalent; e SE = standard error. † Results of nutrition intake from covariance analysis with adjustment for child’s gender and age. (DOCX) [file pone.0123664.s003.docx]

**S 2 Table. Dietary intake of energy, macronutrients, dietary fibre, minerals and vitamins of pre-schoolers of non-picky eating and nit-picking meat groups.**

|  | Non-picky eating | | Nit-picking meat | | *p* value | |
| --- | --- | --- | --- | --- | --- | --- |
|  | Mean | SE ^e^ | Mean | SE ^e^ | Unadjusted | Adjusted ^†^ |
| Energy (kcal) | 1627.65 | 34.65 | 1483.10 | 62.03 | 0.054 | 0.102 |
| Protein (g) | 55.77 | 1.37 | 51.70 | 2.29 | 0.164 | 0.199 |
| Fat (g) | 59.29 | 1.31 | 56.38 | 2.99 | 0.329 | 0.330 |
| Carbohydrate (g) | 225.11 | 5.77 | 201.04 | 9.34 | 0.029 | 0.300 |
| Dietary fibre (g) | 7.61 | 0.28 | 6.47 | 0.52 | 0.066 | 0.126 |
| Vitamin A (μgRE ^b^) | 481.72 | 26.51 | 497.97 | 50.55 | 0.779 | 0.125 |
| Thiamine (mg) | 0.81 | 0.03 | 1.00 | 0.22 | 0.388 | 0.122 |
| Riboflavin (mg) | 0.93 | 0.05 | 0.90 | 0.13 | 0.845 | 0.736 |
| Niacin (mgNE ^c^) | 11.18 | 0.32 | 11.38 | 0.68 | 0.776 | 0.608 |
| Vitamin C (mg) | 63.95 | 2.42 | 65.60 | 4.89 | 0.758 | 0.968 |
| Vitamin E (mgα-TE ^d^) | 19.08 | 0.48 | 18.89 | 1.32 | 0.873 | 0.999 |
| Calcium (mg) | 443.05 | 22.87 | 430.56 | 43.57 | 0.803 | 0.672 |
| Magnesium ^a^ (mg) | 230.54 | 6.72 | 196.47 | 9.05 | 0.015 | 0.045 |
| Iron (mg) | 17.29 | 0.52 | 15.04 | 0.83 | 0.022 | 0.104 |
| Zinc (mg) | 9.33 | 0.45 | 8.06 | 0.40 | 0.156 | 0.176 |
| Copper (mg) | 1.48 | 0.04 | 1.52 | 0.16 | 0.660 | 0.555 |

^a^ indicates significant differences between non-picky eating and picky eating groups (*p* < 0.05).

^b^ Retinol equivalent; ^c^ Niacin equivalent; ^d^ α-Tocopherol equivalent; ^e^ SE = standard error.

^†^ Results of nutrition intake from covariance analysis with adjustment for child’s gender and age.
