# Supplementary material for: Growth and Development in Chinese Pre-Schoolers with Picky Eating Behaviour: A Cross-Sectional Study
Source: PLoS One. 2015 Apr 13;10(4):e0123664. doi: 10.1371/journal.pone.0123664 (PMC4395402; doi:10.1371/journal.pone.0123664)
Supplement: S3 Table — a indicates significant differences between non-picky eating and picky eating groups, p < 0.05. b SE = standard error. † Results of food intake from covariance analysis with adjustment for child’s gender and age. (DOCX) [file pone.0123664.s004.docx]

**S 3 Table. Comparison of intake (g/day) of various groups of food between pre-schoolers in non-picky eating and nit-picking meat groups.**

|  | Non-picky eating | | Nit-picking meat | | *p* value | |
| --- | --- | --- | --- | --- | --- | --- |
|  | Mean | SE ^b^ | Mean | SE ^b^ | Unadjusted | Adjusted ^†^ |
| Cereals ^a^ | 273.69 | 8.35 | 211.72 | 13.62 | 0.001 | 0.004 |
| Vegetables ^a^ | 204.26 | 8.74 | 146.70 | 13.99 | 0.002 | 0.003 |
| Fruits | 183.14 | 7.62 | 164.44 | 15.68 | 0.511 | 0.358 |
| Meat ^a^ | 83.19 | 3.91 | 56.93 | 4.97 | < 0.001 | 0.001 |
| Fish ^a^ | 63.19 | 4.33 | 51.09 | 10.38 | 0.226 | 0.132 |
| Eggs ^a^ | 71.80 | 2.37 | 47.16 | 4.50 | 0.003 | 0.003 |
| Milk | 228.90 | 10.42 | 247.29 | 20.82 | 0.423 | 0.517 |
| Nuts and beans | 79.96 | 5.23 | 72.63 | 9.74 | 0.519 | 0.535 |
| Oils and fats | 22.73 | 1.32 | 25.19 | 2.40 | 0.848 | 0.801 |

^a^ indicates significant differences between non-picky eating and picky eating groups, *p* < 0.05.

^b^ SE = standard error.

^†^ Results of food intake from covariance analysis with adjustment for child’s gender and age.
