# Supplementary material for: Growth and Development in Chinese Pre-Schoolers with Picky Eating Behaviour: A Cross-Sectional Study
Source: PLoS One. 2015 Apr 13;10(4):e0123664. doi: 10.1371/journal.pone.0123664 (PMC4395402; doi:10.1371/journal.pone.0123664)
Supplement: S4 Table — a SE = standard error. (DOCX) [file pone.0123664.s005.docx]

**S 4 Table. Comparison of the micronutrient content in whole blood of pre-schoolers in non-picky and nit-picking meat groups.**

|  | Non-picky eating | | Picky eating | | *p* value |
| --- | --- | --- | --- | --- | --- |
|  | Mean | SE ^a^ | Mean | SE ^a^ |  |
| Calcium (mmol/L) | 1.754 | 0.007 | 1.753 | 0.014 | 0.985 |
| Magnesium (mmol/L) | 1.447 | 0.007 | 1.464 | 0.015 | 0.283 |
| Iron (mmol/L) | 7.019 | 0.039 | 7.774 | 0.080 | 0.405 |
| Zinc (μmol/L) | 76.123 | 0.818 | 80.304 | 2.001 | 0.056 |
| Copper (μmol/L) | 15.470 | 0.151 | 16.062 | 0.364 | 0.136 |

^a^ SE = standard error.
